# The Safety of Embryonic Stem Cell Therapy Relies on Teratoma Removal

**DOI:** 10.18632/oncotarget.434

**Published:** 2012-01-28

**Authors:** Chad Tang, Irving L. Weissman, Micha Drukker

**Affiliations:** Stanford University

Heterotopic transplantation of pluripotent stem cells (PSCs) produces growths known as teratomas (Greek word for monstrous), which consist of a heterogeneous amalgamation of fetal-like tissues. Engraftment of even a few undifferentiated cells is potentially sufficient for teratoma formation. Such an event would have far reaching consequences for the future of this field. Current differentiation protocols produce cultures with unknown degrees of purity and are therefore potentially hazardous. We provide here a succinct overview of current methods and outline challenges for the removal of residual teratoma-initiating cells.

Retrospective and prospective approaches for teratoma depletion have been the subject of significant investigation. Retrospective removal includes standard oncologic treatments of formed tumors via radiation, chemotherapy, and surgery [1]. To avoid the inevitable side effects of oncologic treatments, transplantable cells have been modified with suicide genes prior to engraftment. In this way, grafts can be ablated in the event of uncontrolled growth. This approach has been applied clinically and demonstrated efficacy in abrogating graft-versus-host disease in patients undergoing transplantation of donor T-cells modified with an inducible Caspase-9 gene [2]. Naujok et al. have adapted this approach to PSCs through transduction of a construct where an *OCT4* promoter controls HSV1 thymidine kinase expression, allowing for selective ablation of undifferentiated PSCs upon treatment with of Ganciclovir [3]. Unfortunately, these types of removal will eliminate the desired grafted cells along with teratoma-forming cells.

Prospective depletion of teratoma-initiating cells is advantageous to retrospective removal as such methods prevent initial tumor formation. Two main strategies of prospective removal include treatment with agents specifically cytotoxic to undifferentiated cells and mechanical separation. The first was extensively developed by the lab of Dr. Andre Choo, who produced the cytotoxic antibody mAb 84 which targets PODXL, a protein abundantly expressed by PSCs [4]. Other molecules such as ceramides have also been employed. For example, Bieberich et al. utilized the ceramide analogue, N-oleoyl serinol, to induce selective apoptosis in PSC-derived cultures [5].

Non-cytotoxic cell separation has emerged as a central approach for depletion of teratoma-initiating cells. This method relies on tagging undifferentiated cells either by inducing reporter gene expression or through reversible labeling of surface antigens. Labeled cells are then removed through fluorescence- or magnetic-activated cell sorting (FACS or MACS). Although tagging PSC-specific surface antigens is considered safer than introducing gene reporters [6], this goal has been difficult to achieve due to the lack of exclusive surface markers. To address this shortage, we have recently created a monoclonal antibody specific for the newly identified PSC antigen named stage specific embryonic antigen-5 (SSEA-5). Furthermore, we demonstrated that FACS using SSEA-5 dramatically reduces the teratoma potential of partially differentiated cultures and completely abolishes this potential when SSEA-5 is combined with two additional markers [7]. As an alternative, a recent study demonstrated that MACS employing a lectin that binds PSC-expressed carbohydrates facilitates removal of undifferentiated cells [8].

A major advantage to utilizing surface markers for prospective separation is that this method relies on the intrinsic properties of the cells and therefore can be applied to all PSC lines and differentiation conditions. A typical protocol may entail removing cells expressing a tailored combination of surface markers such as SSEA-5 and/or CD9 and/or CD90 [7]. Such a protocol may occur before full maturation of a culture or with the final cellular product. In cases where cell dissociation perturbs interactions required for graft's function, post-depletion re-aggregation is required. We also note that antibody-based sorting is safer than genetic modification since the latter has been implicated in oncogenic transformation [6].

In summary, we propose that prospective removal is superior to retrospective approaches since the latter may result in anatomical damage and also risks malignant and/or metastastic transformation. As such, retrospective removal should always be considered a backup rather than first line. We also believe that clinical utilization of PSC derivatives should rely on combination of prospective approaches to provide the highest level of safety. One example is to utilize PSC-specific antibodies for cell separation followed by a subsequent cytotoxic antibody incubation step [4]. Ultimately, teratoma removal poises a significant yet surmountable challenge. With development of stringent removal procedures and close monitoring of PSC-derived grafts, we believe that such therapeutics can be applied effectively and safely.
